# Compounds from *Agathis* *dammara* exert hypoglycaemic activity by enhancing glucose uptake: lignans, terpenes and others

**DOI:** 10.1007/s13659-024-00440-4

**Published:** 2024-03-22

**Authors:** Zhe-Wei Yu, Bang-Ping Cai, Su-Zhi Xie, Yi Zhang, Wen-Hui Wang, Shun-Zhi Liu, Yan-Lin Bin, Qi Chen, Mei-Juan Fang, Rong Qi, Ming-Yu Li, Ying-Kun Qiu

**Affiliations:** 1 School of Pharmaceutical Sciences, Xiamen University, Xiamen, 361102 China; 2 School of Basic Medical Sciences, Peking University, Beijing, 100191 China; 3 Xiamen Botanical Garden, Xiamen, 361003 Fujian China; 4 Xiamen Medical College Affiliated Haicang Hospital, Xiamen, 361026 China

**Keywords:** *Agathisdammara*, Lignans, Terpenes, Hypoglycaemic, Glucose uptake

## Abstract

In this study, two new kaurane diterpenes (1**6**, **17**), together with 12 lignans (**1–12**), a triterpene (**15**), and two other compounds (**13**, **14**) were isolated from the woods of *Agathis* *dammara*. The structure of the new compound was determined by HR ESIMS and 1D/2D NMR spectroscopy, and its absolute configuration was determined by electronic circular dichroism (ECD) exciton chirality method. Compounds **5**, **11**, **14** exhibit significant hypoglycaemic activity in zebrafish, and their mechanism of action is to enhance glucose uptake in zebrafish.

**Graphical Abstract:**

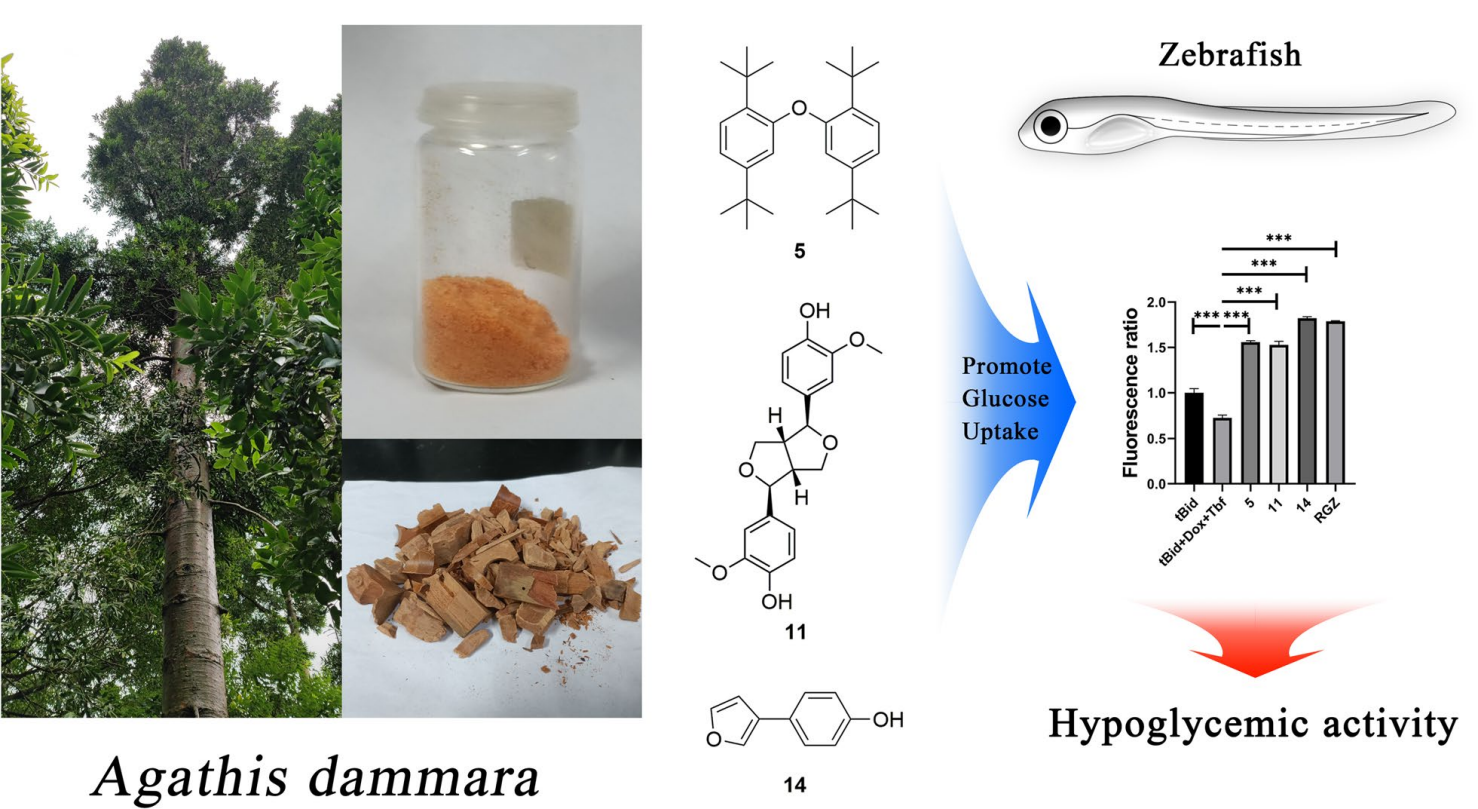

**Supplementary Information:**

The online version contains supplementary material available at 10.1007/s13659-024-00440-4.

## Introduction

**Fig. 1:**
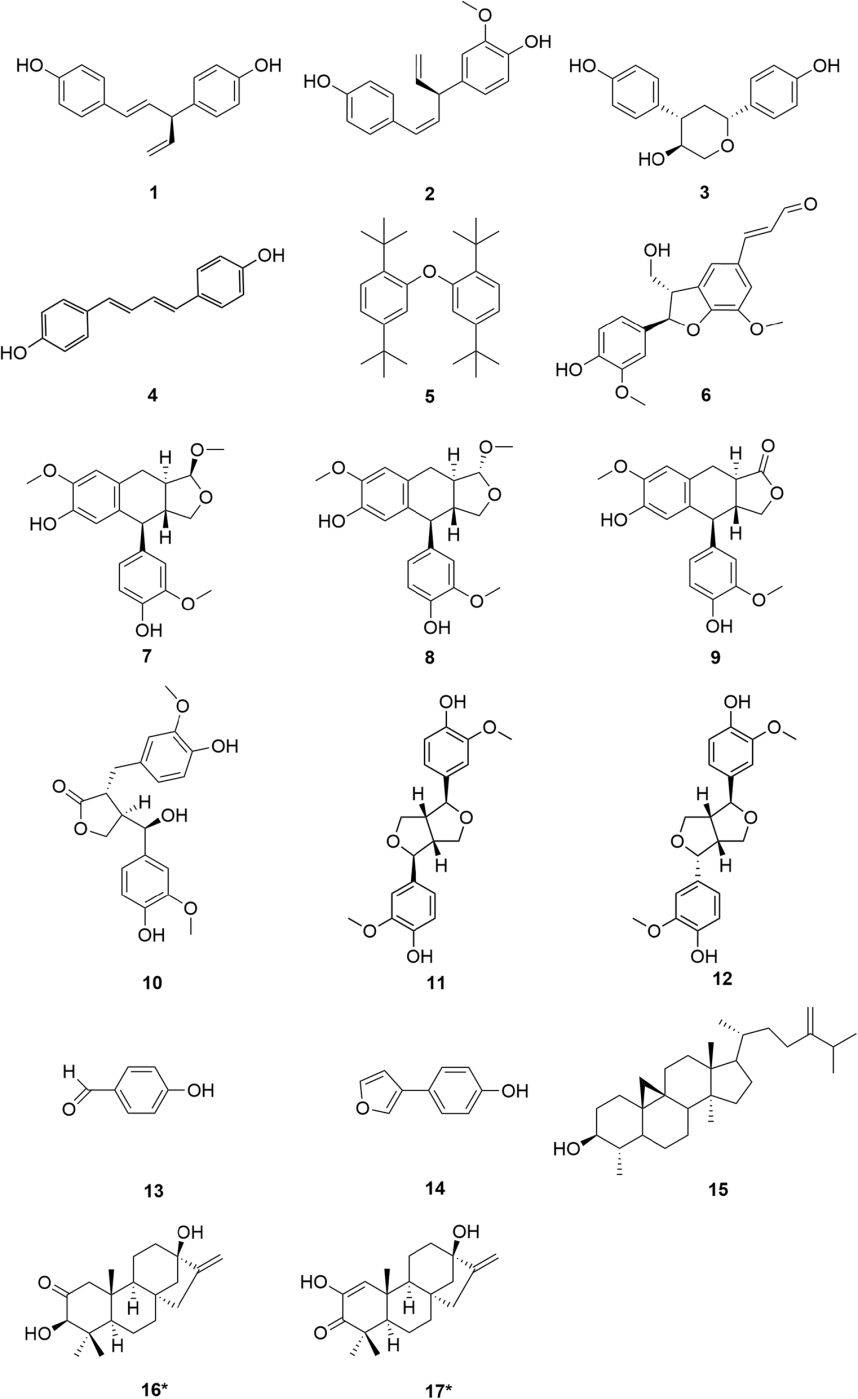
Structure of isolated compounds from the woods of *Agathis* *dammara* (*new compounds)

*Agathis* *dammara* (Lamb.) Rich is a kind of arbor in the family Araucariaceae and the genus *Agathis* [1]. The origin of *A.* *dammara* is Malaysia and the Philippines, and it has been introduced to Xiamen, Quanzhou, Fuzhou and other places in China [1]. The trunk of *A.* *dammara* is rich in resin, which is widely used in industry and medicine [2]. Malaysians use this resin to treat a variety of complex and inflammation-related ailments including arthritis, headaches, muscle pain, burns, fever, diarrhoea, abdominal pain [3]. Related studies have shown that *A.* *dammara* has potential activities of hypoglycaemic, hypolipidemic, antioxidant, anti-inflammatory [2]. In our parallel study, we found that the total diterpene component of *A.* *dammara* and its rich monomeric compound, araucarone could effectively mitigate the inflammatory response in vascular smooth muscle cells, and consequently impede the progression of abdominal aortic aneurysm through suppressing NF-kappaB/NLRP3 pathway activation [4]. In the current study, two new kaurane diterpenes (1**6**, **17**), together with 12 lignans (**1–12**), a triterpene (**15**), and two other compounds (**13**, **14**) were isolated from the woods of *A.* *dammara* (Fig. 1). Presented herein are the isolation, structure identification, hypoglycaemic biological evaluation and biological mechanism studies of these compounds.

## Results and discussion

### Structural identification of new compounds

**Fig. 2:**
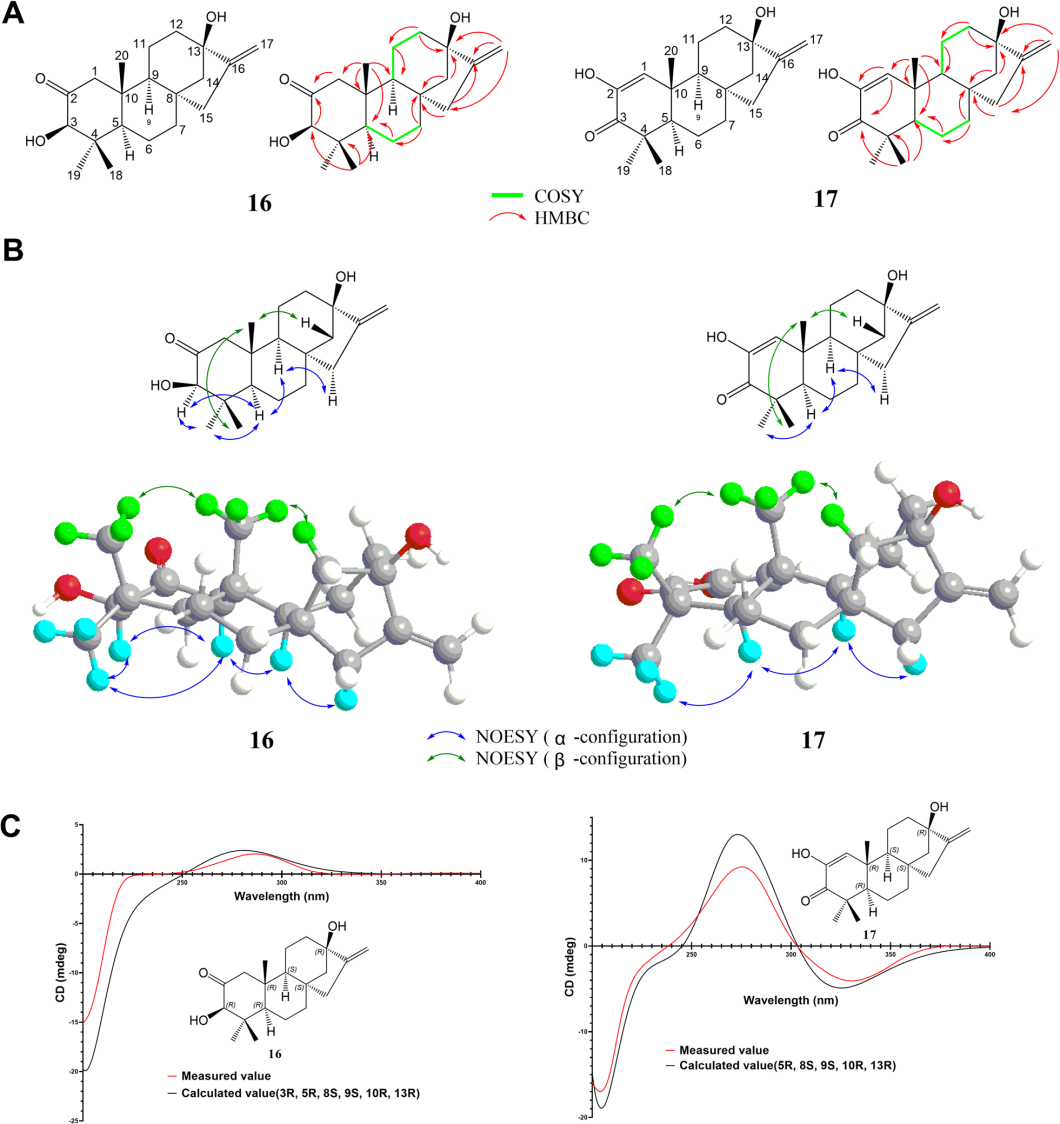
Structural elucidation of compounds **16**, **17**. **A** COSY and key HMBC signals. **B** NOESY correlations and relative configurations. **C** ECD spectral of **16**, **17** versus calculated values

(3β)-3,13-Dihydroxykaur-16-en-2-one (**16**) is white powder. HRESIMS shows that the molecular formula is C_20_H_30_O_3_ (*m/z* 319.2265 [M + H]^+^, calcd 319.2268), and the degree of unsaturation is 6. UV spectra indicated carbon–carbon double bond (λ_max_ = 210 nm), IR spectra indicated carbonyl (σ_min_ = 1712 cm^−1^) and hydroxyl (σ_min_ = 3424 cm^−1^). There were 3 methyl signals in ^1^H-NMR of **16** at *δ*_H_ 0.70 (br s, 3H, H-18), 1.19 (s, 3H, H-19), and 0.97 (s, 3H, H-20). In addition, two alkene hydrogen signals at *δ*_H_ 4.85 (m, H-17), 5.00 (m, H-17), and one methine bearing to hydroxyl at *δ*_H_ 3.87 (br s, H-3) were found. In the ^13^C-NMR of **16**, the signal at *δ*_C_ 210.7 (C-2) suggested the presence of carbonyl. The DEPT spectrum showed 3 primary carbons, 8 secondary carbons, 3 tertiary carbons, and 6 quaternary carbons, including a quaternary carbon connected to a hydroxyl at *δ*_C_ 80.0 (C-13). The methyl remote coupling signal of HMBC can confirm that the *δ*_H_ 0.70 (br s, CH_3_-18), 1.19 (s, CH_3_-19) connecting to *δ*_C_ 45.3 (C-4) then to 82.8 (C-3) and 54.4 (C-5), and *δ*_H_ 0.97 (s, CH_3_-20) connecting to *δ*_C_ 45.2 (C-10) then to 53.2 (C-1), 54.4 (C-5) and 54.0 (C-9). The coupling from *δ*_H_ 3.87 (br s, H-3), 2.03; 2.64 (m; dd, H-1) and *δ*_C_ 210.7 (C-2) confirmed the position of carbonyl at C-2. In the HMBC spectrum, correlations could be observed from *δ*_H_ 4.85; 5.00 (m; m, H-17) at the end side of the alkene to *δ*_C_ 155.3 (C-16), and then to the quaternary carbon 80.0 (C-13) and methylene 47.1 (C-15). Moreover, the HMBC correlations showed that there is an extra D ring in the closure of C-8, 13, 14, 15, and 16, which proves that this is a tetracyclic diterpene (Fig. 2A). **16** is very similar to the structure of the known compound excoecarin K [5]. Excoecarin K is an enantio-kaurane tetracyclic diterpene, indicating that **16** should also be a tetracyclic diterpene of the kaurane skeleton. The difference between **16** and excoecarin K is that C-13 of **16** has a higher chemical shift (*δ*_C_ 43.7 to 80.0) because 13-H is replaced by 13-OH. In NOESY, there are related signals of *δ*_H_ 0.97 (s, CH_3_-20), 0.70 (br s, CH_3_-18), and 2.04 (m, H-14β), which can indicate that C-14 on the C ring is tilted in the same direction as CH_3_-20, CH_3_-18; *δ*_H_ 1.19 (s, CH_3_-19), there are also correlation signals between 1.49 (m, H-5), 1.29 (br s, H-9), 3.87 (br s, H-3), and 2.12 (br d, H-15α), indicating that the D ring is tilted in the direction opposite to that of C-14, that is, the configurations of C-15, C-16 and CH_3_-19, H-5 and H-9 are consistent, that is, CH_3_-20, CH_3_-17, CH_3_-18, 3-OH, 13-OH are β configurations, H-5, H-9, C-15, and C-16 are in α configuration (Fig. 2B). The data determined by ECD showed that the absolute configuration of the compound is 3*R*, 5*R*, 8*S*, 9*S*, 10*R*, and 13*R* (Fig. 2C).

By comparison the ^13^C-NMR signals of **17** and **16**, the difference between them leis in A ring. The C-3 position changed from a hydroxyl connected methine to a carbonyl, which shifted from *δ*_C_ 82.8 to *δ*_C_ 201.0. In addition, the -CH_2_CH(OH)- moiety at C-1, 2 position of **16** with signals at *δ*_C_ 53.2, 210.7 changes to a hydroxyl connected alkene double bone (*δ*_C_ 128.4 and 144.3) to form an α,β-unsaturated ketone structure. on the aids of HSQC, COSY and HMBC, the planar structure could be established as shown in Fig. 2A. The comparison of NOE correlations (Fig. 2B), optical rotation, ECD spectrum and calculated value showed that the absolute configuration of **17** is consistent with **16** (Fig. 2C).

Other isolated compounds were identified from their NMR and MS data, as well as by comparing these data with reported values, and they were: hinokiresinol (**1**) [6], methoxynyasol (**2**) [7], sugiresinol (**3**) [8], bis (4-hydroxyphenyl) buta-1,3-diene (**4**) [9], 2,2'-oxybis(1,4)-di-tertbutylbenzene (**5**) [10], balanophonin (**6**) [11], todolactol C (**7**) [12], (1*R*,3a*R*,4*S*,9a*R*)-1,7-dimethoxy-4-(4'-hydroxy-3'-methoxyphenyl)-1,3,3a,4,9,9a-hexahydronaphtho[2,3-c]furan-6-ol (**8**) [12], α-conidendrin (**9**) [13], (7'*R*)-hydroxymatairesanol (**10**) [14], pinoresinol (**11**) [15], epipinoresinol (**12**) [16], *p*-hydroxybenzaldehyde (**13**), 4-(3-furanyl)phenol (**14**), and cycloeucalenol (**15**) [17].

## Study of hypoglycaemic activity of representative compounds

The zebrafish (*Danio* *rerio*) has similar glucose metabolism pathways and metabolites with those of humans, which made it an ideal model for studying the hypoglycaemic activity of drugs [18, 19]. In this study, a *Tg* *(Ins:htBid* ^*TE−ON*^*;LR)* pancreatic β cells ablation zebrafish model was used to induce hyperglycaemic symptoms [20]. In the transgenic zebrafish, the truncated human Bid protein (htBid) was derived by an insulin promoter and controlled under the tetracycline- and ecdysone-inducible system. After induction with doxycycline (Dox) and tebufenozide (Tbf), the proapoptotic tBid expression in β cells resulted in the apoptosis of β cells, which were labelled by the transgenic line *Tg* *(− 1.2ins:H2Bmcherry)*. The zebrafish was then incubated with compounds **1–17** (5 μmol/L) for 24 h as biological activity testing.

**Fig. 3:**
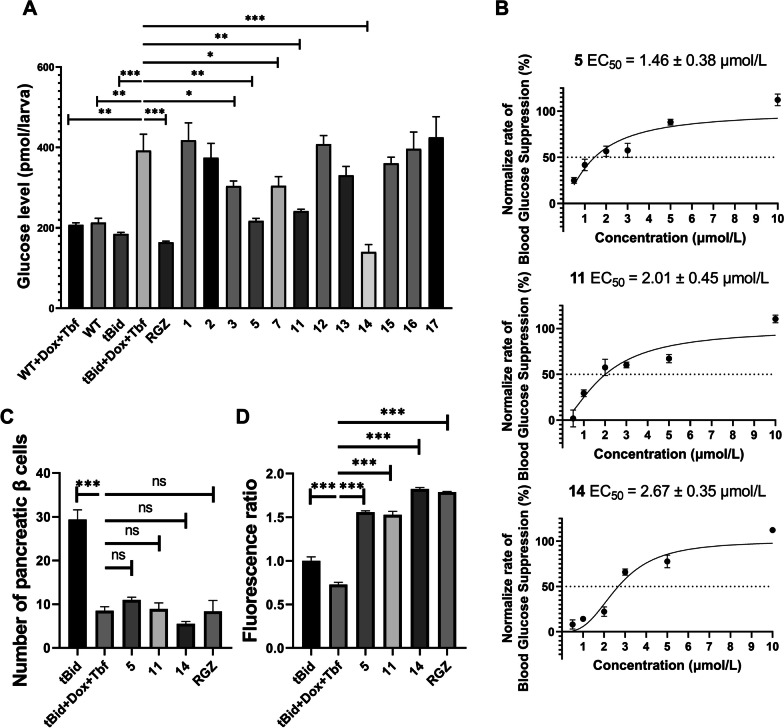
Biological evaluation and mechanism study of the hypoglycaemic activity of isolated compounds. **A** Blood glucose value of hyperglycaemic zebrafish model after treatment with compounds **1–17** for 24 h. **B** ED_50_ value calculation of the hypoglycaemic activity of compounds **5**, **11**, **14**. **C** Changes in the number of pancreatic β cells in the hyperglycaemic zebrafish model after treatment with compounds **5**, **11**, **14** for 24 h. **D** Changes in the 2-NBDG uptake ability of the hyperglycaemic zebrafish model after treatment with compounds **5**, **11**, **14** for 24 h. (*p < 0.05; **p < 0.01; ***p < 0.001)

The free glucose level was significantly increased after the β cells’ ablation compared with the non-induced transgenic zebrafish, suggesting that the β cells’ ablation caused hyperglycaemia in the zebrafish model. Compounds **3**, **5**, **7**, **11**, **14** reduced glucose levels in the hyperglycaemic zebrafish, and compounds **5**, **11**, **14** had the most significant effect (p < 0.001) (Fig. 3A). We then tested the ED_50_ values of the hypoglycaemic activities of **5**, **11**, **14**, which were 1.46 ± 0.38, 2.01 ± 0.45, 2.67 ± 0.35 μmol/L respectively (Fig. 3B).

Inhibitory of blood glucose elevation mainly includes two mechanisms in different tissues: promotion of glucose uptake in peripheral tissue and regeneration of pancreatic β cells. Two zebrafish modes were applied to reveal the hypoglycemic mechanism.

Double transgenic zebrafish *Tg* *(Ins:htBid*^*TE−ON*^*;* *LR);* *Tg* *(Ins:H2Bmcherry)* was applied to monitor the number of β cells after the treatment of compounds. The *Tg* *(Ins:H2Bmcherry)* zebrafish pulls red fluorescent protein (mCherry) to be expressed in the β cells under the regulation of an insulin-specific promoter and is used to label the β cells. Therefore, zebrafish pancreatic β cells can be observed, statistically and imaged through fluorescence microscopy [19]. Compared with the β cells ablation group (tBid + Dox + Tbf + DMSO), the number of pancreatic β cells in the hyperglycaemic zebrafish model did not change after 24 h of treatment with compounds **5**, **11**, **14** (Fig. 3C). This suggests that the hypoglycaemic activity of **5**, **11**, **14** is independent of pancreatic β cell regeneration.

Then we use the 2-NBDG, a glucose fluorescence analogue, was added in the zebrafish culture medium for indication of glucose uptake [21]. Experimental results show that compounds **5**, **11**, **14** can significantly increase the uptake of 2-NBDG in hyperglycaemic zebrafish, with similar activity of the positive drug rosiglitazone (RGZ). This indicates that compounds **5**, **11**, **14** can enhance the blood glucose uptake ability of hyperglycaemic zebrafish, which is the mechanism they exert hypoglycaemic activity (Fig. 3D).

## Experimental section

### General experimental procedures

Optical rotations were measured on a JASCO P-200 polarimeter (Tokyo, Japan). The UV spectra were recorded in methanol using a Shimadzu UV-260 spectrophotometer (Kyoto, Japan). The ECD spectra were measured by a JASCO J-810 spectrometer (Tokyo, Japan). The IR measurements were performed on a Perkin-Elmer 683 spectrometer. The NMR experiments were conducted on Bruker Avance III-600 MHz spectrometers in CDCl_3_ and DMSO-*d*_6_. The HR ESIMS data were acquired using a Thermo Fisher Q-Exactive mass spectrometer (Boston, USA). Column chromatography (CC) separations were carried out by using silica gel (300–400 mesh; Qingdao Haiyang Chemical Co., Ltd., Qingdao, China), and ODS RP-C_18_ (40–63 μm, FuJi, Aichi, Japan). An Agilent 1260 series system (California, USA) with an COSMOSIL 5C_18_-MS-II (5 μm, 4.6 mm i.d. × 150 mm, Kyoto, Japan) column was used for HPLC analysis. Preparative HPLC was carried out using a Welch Sail 1000 series instrument equipped with a Welch Ultimate XB-C18 column (5 μm, 250 mm × 21.2 mm i.d., China).

### Plant material

Dried wood of *Agathis* *dammara* (Lamb.) Rich was obtain from Xiamen Garden Botanical Garden (24° 26′ 54.6ʺ N 118° 06′ 26.4ʺ E) in March 2021, Xiamen, Fujian Province, China. A voucher specimen (2021BKS) was preserved in School of Pharmaceutical Sciences, Xiamen University, and identified by Associate Professor Quan-Cheng Chen.

## Extraction and isolation

A total of 1 kg of dried *A.* *dammara* wood was extracted and refluxed for 3 times with 2 L of 95% ethanol for 1.5 h to concentrate to obtain 367 g of extract, which was suspended in water and then extracted with the same volume of dichloromethane to obtain 100 g of dichloromethane extract (Fr. A), first separated by silica gel column (petroleum ether-ethyl acetate 100:1–0:1), divided into 7 components (Fr. A1-Fr. A7).

After HPLC–DAD and TLC analysis, the scheme for further separation of Fr. A2-Fr. A6 was determined, that is, off-line separation by combining reversed-phase and normal-phase open chromatographic columns, combined with preparative HPLC. Each component was firstly passed through Chromatorex C_18_ as the chromatographic stationary phase, and carried out gradient elution using the conditions explored by HPLC analysis (MeOH–Water 5–100%). The eluted components were analyzed by TLC, using dichloromethane and methanol as developing solvents, and using a developing solvent formula with an Rf value of 0.2–0.3 on TLC as the mobile phase for silica gel column chromatography separation. The purity of the components separated at this time was checked by TLC and HPLC, and if impure, they were further purified by preparative HPLC (Additional file 1: Fig. S1).

### Spectra data of the new compounds

(3β)-3,13-dihydroxykaur-16-en-2-one (**16**) is white powder, [α]20 D = 54°, melting point is 78.6 ~ 79.0 ℃. HR ESIMS shows that the molecular formula is C_20_H_30_O_3_ (*m/z* 319.2265 [M + H]^+^, calcd 319.2268), and the degree of unsaturation is 6. UV spectra indicated carbon–carbon double bond (λ_max_ = 210 nm), IR spectra indicated carbonyl (σ_min_ = 1712 cm^−1^) and hydroxyl (σ_min_ = 3424 cm^−1^).

2,13-dihydroxykaura-1,16-dien-3-one (**17**) is white powder, [α]20 D = 24°, melting point is 118.8 ~ 121.2 ℃. HR ESIMS showed that the molecular formula is C_20_H_28_O_3_ (*m/z* 317.2108 [M + H]^+^, calcd 317.2111), and the degree of unsaturation is 7. UV spectra indicated carbon–carbon double bond (λ_max_ = 210 nm) and α, β-unsaturated ketone (λ_max_ = 270 nm), IR spectra indicated carbonyl (σ_min_ = 1711 cm^−1^), hydroxyl (σ_min_ = 3425 cm^−1^) structure. Their NMR data are listed in Table 1.

**Table 1:** ^13^C-NMR (150 MHz, CDCl_3_) and ^1^H-NMR (600 MHz, CDCl_3_) spectrum data of compounds** 16** and** 17**

No.	**16**		**17**		Excoecarin K
	*δ*_H_ (mult., *J* in Hz)	*δ*_C_	*δ*_H_ (mult., *J* in Hz)	*δ*_C_	*δ*_C_
1	2.03 m; 2.64, dd (12.90, 2.00)	53.2	6.45 m	128.4	53.4
2		210.7		144.3	211.0
3	3.87, br s	82.8		201.0	82.8
4		45.3		44.0	45.3
5	1.49, m	54.4	1.68 m	53.9	55.4
6	1.45, m; 1.72, m	20.1	1.55 m; 1.58 m	20.4	20.1
7	1.61, 1.60 m	40.6	1.62 m; 1.65 m	40.6	39.2
8		41.7		41.8	45.5
9	1.29, br s	54.0	1.28 s	49.4	55.4
10		45.2		40.0	44.3
11	1.66, m; 1.67, m	20.4	1.92 m; 1.77 m	20.6	18.4
12	1.58, m; 1.80, m	39.0	1.61 m; 1.82 m	39.0	32.8
13		80.0		80.0	43.7
14	1.28, m; 2.04, m	46.4	2.11 dd (11.0, 2.7);1.33 dd (11.0, 2.7)	47.4	40.5
15	2.12, br d (17.1); 2.27, dd (17.1, 2.0)	47.1	2.11 m;2.27 m	47.3	48.7
16		155.3		155.3	154.9
17	4.85, m; 5.00, m	103.5	4.87 s; 5.03 t (2.6)	103.7	103.6
18	0.70, br s	16.6	1.14 s	21.5	18.6
19	1.19, s	29.7	1.21 s	27.7	29.7
20	0.97, s	18.6	1.30 s	22.0	16.4

### ECD calculations

Conformational analyses were performed by Schrodinger Maestro 12.8 using Conformational Search function. The output conformers were then optimized at OPLS4 force field and filtered by RMSD threshold of 0.1 Å and energy window of 5.02 kcal/mol. The conformations calculated according to the Boltzmann equation at room temperature accounted for less than 1% were removed, and the five conformations with the energy closest to the lowest energy conformation were screened to calculate the excitation spectrum. The theoretical calculations were carried out using Gaussian 09W. The chosen conformers were finally optimized at B3LYP/6-31G(d) in methanol.

The ECD calculations of the chosen conformers were executed in methanol with Time-dependent Density functional theory (TD-DFT) at B3LYP/6–31 + G(d) or B3LYP/6–311 + G(d,p) level by Gaussian 09W. Rotatory strengths for 20 or 30 excited states were calculated. Finally, GaussView 6.0 was used to add the spectrum according to the energy of various conformations according to the Boltzmann energy equation to obtain the ECD calculation spectrum.

### Establishment and treatment of hyperglycemic zebrafish model

Fertilized eggs of zebrafish strain *Tg* *(Ins:htBid*^*TE−ON*^*,* *Ins:H2Bmcherry)* were collected the next day, and this time was recorded as day 0 post fertilization (0 dpf). Tbf (3 μL, 50 mmol/L) and Dox (3 μL, 100 mmol/L) were added into a 3.5 cm cell culture dish containing 6 mL 0.3 × Danieau’s buffer. Then, the dish was placed in a zebrafish incubator without light for 48 h to induce β cells in 2 dpf ablation.

Diabetic zebrafish that were ready to be used in experiments after β cells ablation were rinsed with 0.3 × Danieau’s buffer to remove Tbf and Dox. The larvae were placed into a 24-well plate at a density of 10 zebrafishes/well, in 2 mL of egg water. All compounds were made in 10 mM with DMSO (prepare other concentrations when calculating ED_50_). For the treatment, each group was added accordingly with 2 mL of egg water treated with 1 μL of each of the compounds (10 mmol/L) to reach the final concentration of (5 μmol/L), and the control group was treated with the same amount of DMSO. The treatments lasted for 24 h in the zebrafish incubator. The group of tBid which was not induced with Tbf and Dox was used as a control of normal zebrafish.

### Total glucose level test

After the compound treatment, a pool of 10 larvae was homogenized in 100 μL of sample buffer. The homogenate was spun at 10,000 rpm for 10 min. Free glucose in 10 μL of supernatant (equivalent of one larva) was determined according to the manufacturer’s instructions. Fluorescence (excitation, 520 nm; emission, 580–640 nm) was measured using a SpectraMax M5 Microplate Reader (Molecular Devices, California, USA). Each sample was measured for three pools. The obtained data were imported into GraphPad Prism 8.0.2 software, and the statistical differences among the groups were analyzed using the t-test method.

### 2-NBDG test

After the compound treatment, zebrafish (6 dpf) were incubated in a culture medium containing 600 μmol/L 2-NBDG (Apexbio, B6035, Texas, USA) for 3 h. A pool of 5 larvae was homogenized in 100 μL of sample buffer. The homogenate was spun at 10,000 rpm for 10 min. The supernatant (30 μL) was placed into a 96-well plate to detect fluorescence (excitation, blue 475 nm; emission, 500–550 nm) using a SpectraMax M5 Microplate Reader (Molecular Devices, California, USA). Each sample was measured for three pools. The obtained data were imported into GraphPad Prism 8.0.2 software, and the statistical differences among the groups were analyzed using the ordinary one-way (ANOVA) method.

### Microscopic imaging and counting of pancreatic cells

The compounds with 0.3 × Danieau’s buffer were washed out after the compound treatment in 24 h. The larvae with 4% paraformaldehyde were fixed overnight at 4 °C and placed on a slide with aqua-mount (Richard-AllanScientific, Michigan, USA) with the right sides of the larvae facing up to expose the islets. The number of cells number was counted according to the RFP and GFP under a Leica TCS SP8 microscope (Leica, Weztlar, Germany) with 63 × lens. Microscopic images were processed and counted by LASX Office 1.4.5 software. The obtained data were imported into GraphPad Prism 8.0.2 software, and the statistical differences among the groups were analyzed using the ordinary one-way (ANOVA) method.

## Conclusion

In summary, two new kaurane diterpenes (3β)-3,13-dihydroxykaur-16-en-2-one (**16**) and 2,13-dihydroxykaura-1,16-dien-3-one (**17**) along with 15 known compounds were isolated from the woods of *A.* *dammara*. Among them, three compounds **5**, **11**, and **14** showed significant hypoglycaemic activity in the hyperglycaemic zebrafish model. Further mechanism analysis showed that this activity was caused by improving the glucose uptake ability of zebrafish. These findings enrich the structural diversity of diterpenes from the genus *Agathis* and provide potential lead compounds for the development of naturally derived antihyperglycemic drugs.

### Supplementary Information

**Additional file 1:** Spectral data, mass data and calculation details of new compounds, and flow diagram of compounds separation.

## Data Availability

The datasets used or analysed during the current study are available from the corresponding author on reasonable request.
