# Supplementary material for: Compounds from Agathis dammara exert hypoglycaemic activity by enhancing glucose uptake: lignans, terpenes and others
Source: Nat Prod Bioprospect. 2024 Mar 22;14(1):23. doi: 10.1007/s13659-024-00440-4 (PMC10959857; doi:10.1007/s13659-024-00440-4)
Supplement: Supplementary file 1 — Additional file 1: Spectral data, mass data and calculation details of new compounds, and flow diagram of compounds separation. [file 13659_2024_440_MOESM1_ESM.docx]

Compounds from *Agathis Dammara* Exert Hypoglycemic Activity by Enhancing Glucose Uptake: Lignans, Terpenes and Others

*Zhe-Wei Yu*^1,2,§^ *Bang-Ping Cai^3,^*^§^*,* *Su-Zhi Xie*^4^, *Yi Zhang*^1^, *Wen-Hui Wang*^1^, *Shun-Zhi Liu*^1^, *Yan-Lin Bin*^1^, *Qi Chen*^1^, *Mei-Juan Fang*^1^*, Rong Qi*^2^, *Ming-Yu Li*^1,^*, *Ying-Kun Qiu*^1,^*

^1^ School of Pharmaceutical Sciences, Xiamen University, Xiamen 361102, China

^2^ School of Basic Medical Sciences, Peking University, Beijing 100191, China

^3^ Xiamen Botanical Garden, Xiamen, Fujian 361003, China;

^4^ Xiamen Medical College Affiliated Haicang Hospital, Xiamen, 361026, China.

^§^ These authors contributed equally

* Corresponding Authors

E-mails: [qyk@xmu.edu.cn](mailto:qyk@xmu.edu.cn) (Ying-Kun Qiu), [limingyu@xmu.edu.cn](mailto:limingyu@xmu.edu.cn) (Ming-Yu Li)

Tel: [+86](mailto:+86) 13656035105 (Ying-Kun Qiu), [+86](mailto:limingyu@xmu.edu.cn) 18659242557 (Ming-Yu Li)

List of Contents

[**Figure S1** Isolation flow chart of compounds **1-17** 3](#_Toc156571019)

[**Figure S2** Quasi-molecular ion peak signal in HRESI-MS spectra of **16** 4](#_Toc156571020)

[**Figure S3** UV spectra of **16** 5](#_Toc156571021)

[**Figure S4** IR spectra of **16** 6](#_Toc156571022)

[**Figure S5** ^1^H NMR spectra of **16** 7](#_Toc156571023)

[**Figure S6** ^12^C NMR spectra of **16** 8](#_Toc156571024)

[**Figure S7** DEPT 135 spectra of **16** 9](#_Toc156571025)

[**Figure S8** HSQC spectra of **16** 10](#_Toc156571026)

[**Figure S9** HMBC spectra of **16** 11](#_Toc156571027)

[**Figure S10** COSY spectra of **16** 12](#_Toc156571028)

[**Figure S11** NOESY spectra of **17** 13](#_Toc156571029)

[**Figure S12** Quasi-molecular ion peak signal in HRESI-MS spectra of **17** 14](#_Toc156571030)

[**Figure S13** UV spectra of **17** 15](#_Toc156571031)

[**Figure S14** IR spectra of **17** 16](#_Toc156571032)

[**Figure S15** ^1^H NMR spectra of **17** 17](#_Toc156571033)

[**Figure S16** ^12^C NMR spectra of **17** 18](#_Toc156571034)

[**Figure S17** DEPT 135 spectra of **17** 19](#_Toc156571035)

[**Figure S18** HSQC spectra of **17** 20](#_Toc156571036)

[**Figure S19** HMBC spectra of **17** 21](#_Toc156571037)

[**Figure S20** COSY spectra of **17** 22](#_Toc156571038)

[**Figure S21** NOESY spectra of **17** 23](#_Toc156571039)

[**Table S1** Energies and boltzmann distribution information of each conformation of compound **16** for ECD calculation 24](#_Toc156571040)

[**Table S2** Energies and boltzmann distribution information of each conformation of compound **17** for ECD calculation 24](#_Toc156571041)

[**Table S3** Cartesian coordinates of new compound**s** for ECD calculation 25](#_Toc156571042)

## Isolation flow chart of compounds **1-17**


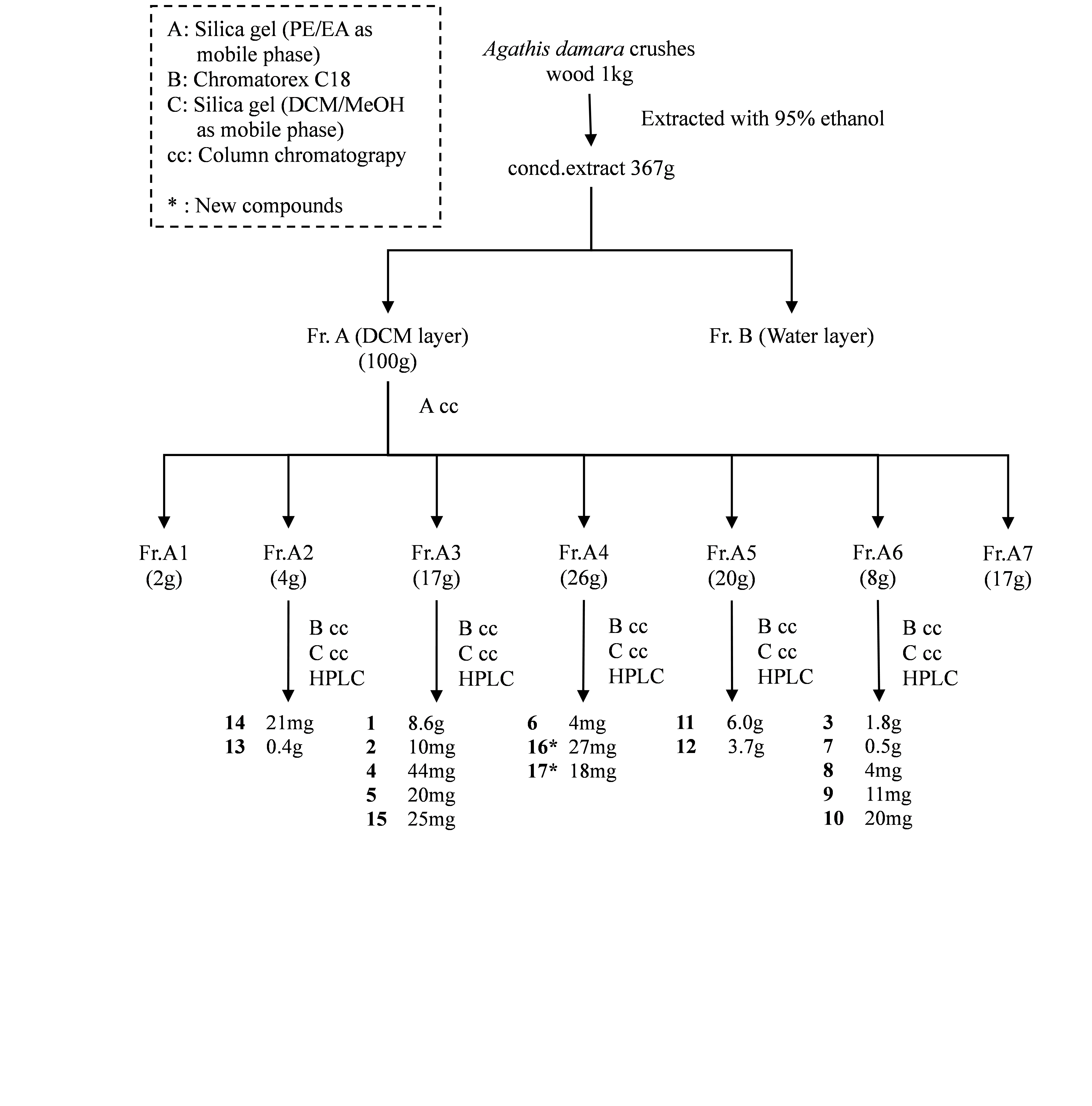


## Quasi-molecular ion peak signal in HRESI-MS spectra of **16**


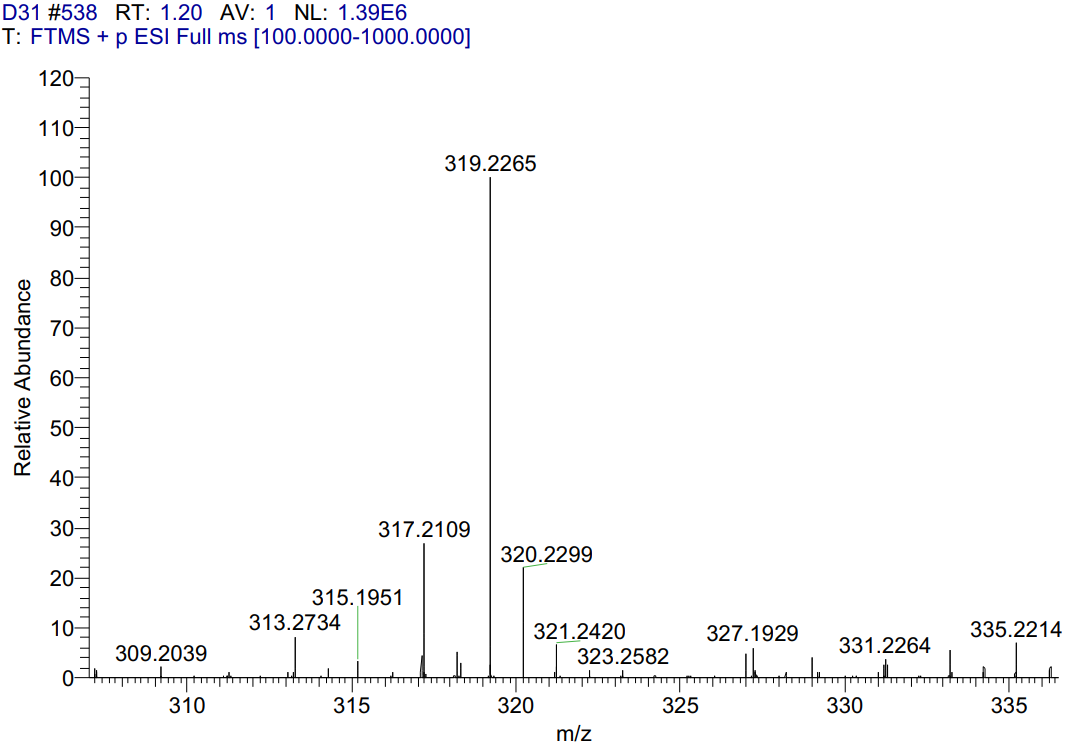


## UV spectra of **16**


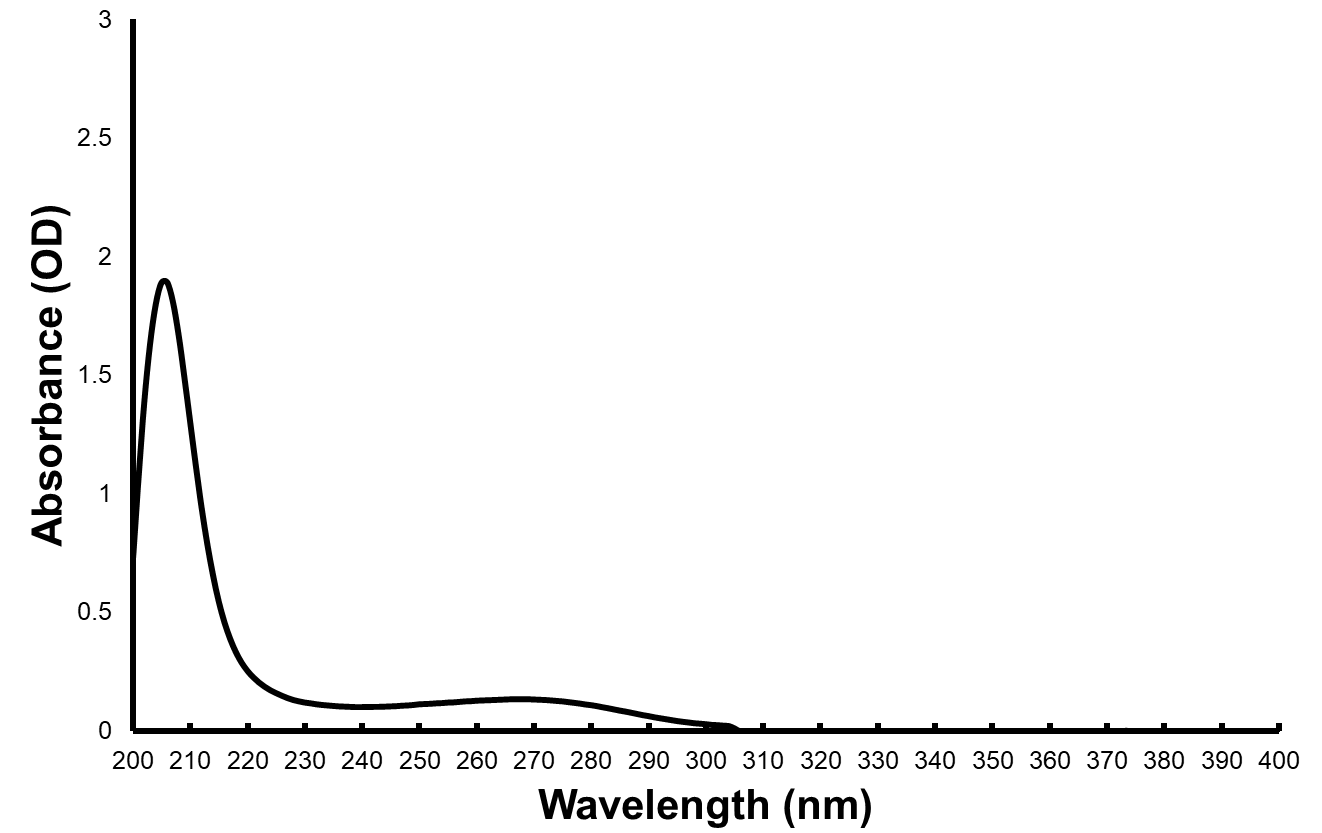


## IR spectra of **16**

**
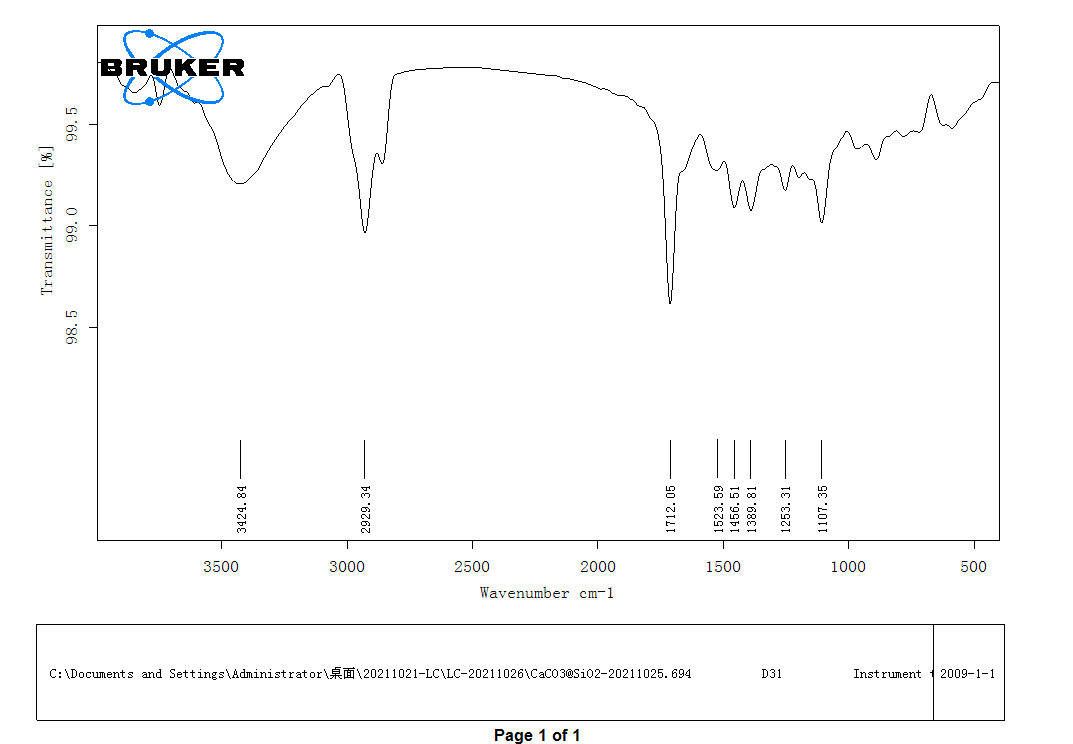
**

## ^1^H NMR spectra of **16**

## ^12^C NMR spectra of **16**

## DEPT 135 spectra of **16**

## HSQC spectra of **16**

## HMBC spectra of **16**

## COSY spectra of **16**

## NOESY spectra of **17**

## Quasi-molecular ion peak signal in HRESI-MS spectra of **17**


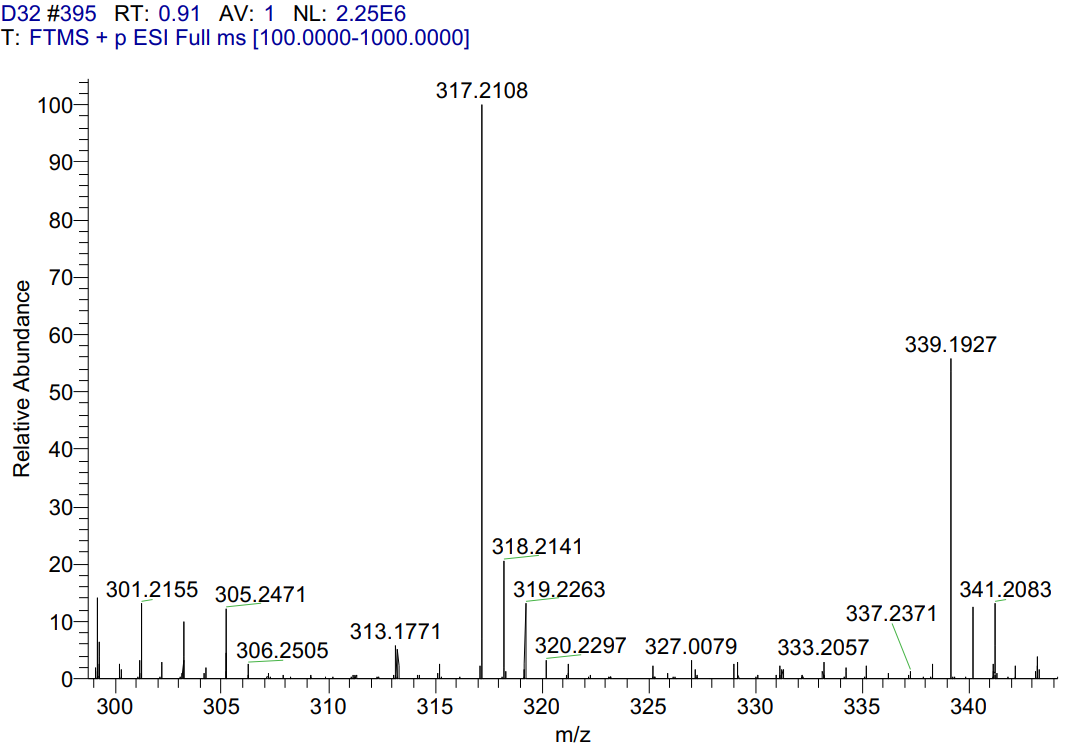


## UV spectra of **17**

**
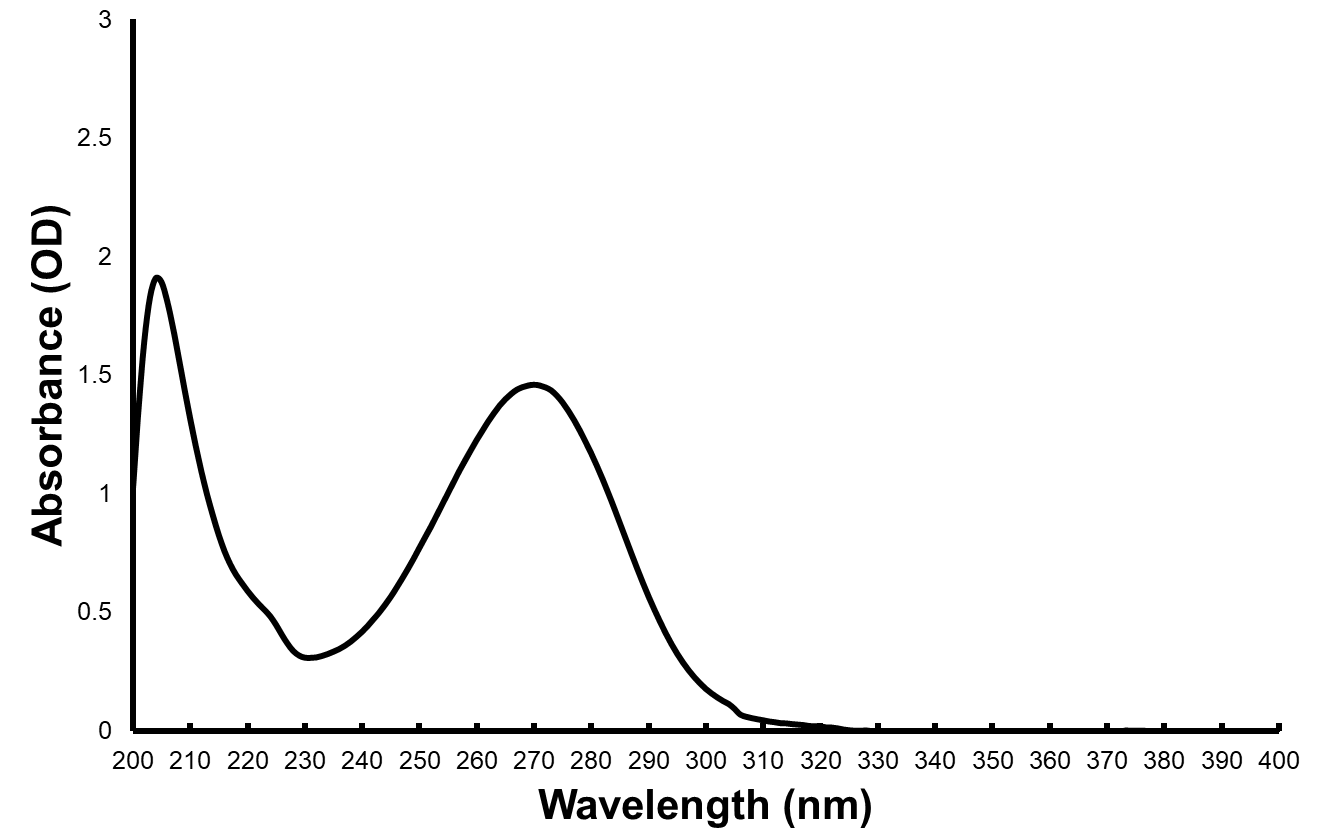
**

## IR spectra of **17**

**
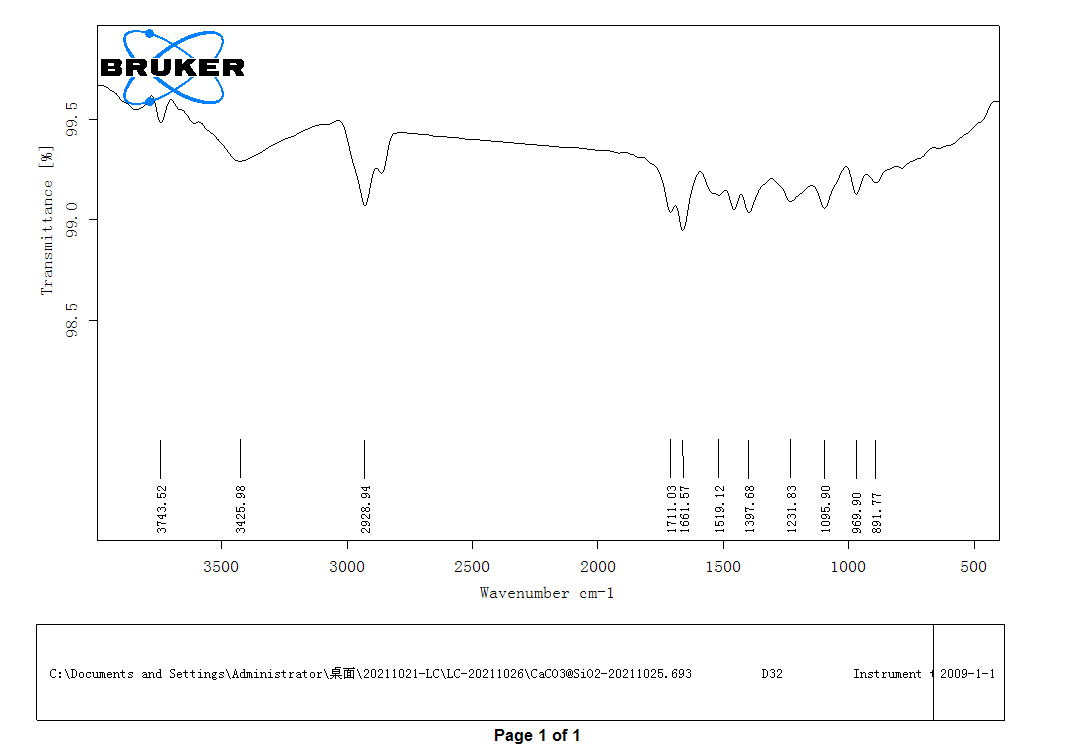
**

## ^1^H NMR spectra of **17**

## ^12^C NMR spectra of **17**

## DEPT 135 spectra of **17**

## HSQC spectra of **17**

## HMBC spectra of **17**

## COSY spectra of **17**

## NOESY spectra of **17**

### Energies and boltzmann distribution information of each conformation of compound **16** for ECD calculation

| Comformation No. | Energy (hartree) | Boltzmann pop. |
| --- | --- | --- |
| 1 | -1005.716513 | 0.374319 |
| 2 | -1005.715065 | 0.080812 |
| 3 | -1005.715155 | 0.088881 |
| 4 | -1005.716514 | 0.374799 |
| 5 | -1005.715069 | 0.081189 |

### Energies and boltzmann distribution information of each conformation of compound **17** for ECD calculation

| Comformation No. | Energy (hartree) | Boltzmann pop. |
| --- | --- | --- |
| 1 | -1004.520178 | 0.691299 |
| 2 | -1004.518745 | 0.151465 |
| 3 | -1004.518780 | 0.157236 |

### Cartesian coordinates of new compound**s** for ECD calculation

Conformation 1 of **16**

C 1.39579 -1.56295 -0.86006

C 2.85382 -1.56025 -0.48919

C 3.59258 -0.24845 -0.68225

C 2.90996 0.89499 0.14558

C 1.3756 0.88053 -0.18782

C 0.60995 -0.48654 -0.03959

C 0.5782 2.01834 0.47629

C -0.7722 2.20134 -0.22476

C -1.63746 0.93393 -0.25101

C -0.80172 -0.30231 -0.72531

C -2.38079 0.64692 1.07866

C -3.45547 -0.36666 0.63768

C -2.76119 -1.70527 0.33694

C -1.69936 -1.57585 -0.77431

C 3.53744 2.21912 -0.34815

C 3.25804 0.74642 1.64158

O 4.96044 -0.40899 -0.37128

H 1.34147 1.09197 -1.26979

O -4.45995 -0.63978 1.60844

C 0.51883 -1.01321 1.41227

H -0.54634 -0.07991 -1.77179

O 3.44611 -2.52562 -0.02032

C -3.97515 0.27143 -0.64898

C -2.85544 1.12852 -1.2148

C -5.21358 0.12797 -1.121

H 1.31649 -1.33194 -1.93128

H 1.00073 -2.56893 -0.69789

H 3.49758 0.02113 -1.74685

H 0.4288 1.82053 1.54498

H 1.13496 2.95857 0.41572

H -1.33983 3.01513 0.24629

H -0.58124 2.5175 -1.2613

H -1.76011 0.26122 1.88904

H -2.86238 1.56943 1.43425

H -2.32643 -2.0716 1.27487

H -3.51912 -2.43571 0.02935

H -2.22964 -1.56219 -1.73363

H -1.09 -2.48468 -0.79451

H 3.22903 2.45145 -1.37486

H 3.26146 3.06434 0.29007

H 4.6289 2.13675 -0.33118

H 3.03533 -0.24388 2.04469

H 2.71198 1.48293 2.23992

H 4.32749 0.92209 1.78785

H 5.02388 -1.31144 0.00301

H -4.96185 0.18263 1.73727

H -0.22952 -1.80698 1.48464

H 0.25611 -0.23846 2.13528

H 1.46343 -1.45402 1.73989

H -3.15442 2.18575 -1.20336

H -2.60904 0.88455 -2.25541

H -5.9392 -0.4934 -0.60151

H -5.54833 0.62417 -2.02918

Conformation 2 of **16**

C 1.3923 -1.55917 -0.86972

C 2.84998 -1.56077 -0.49731

C 3.59107 -0.24914 -0.68273

C 2.90934 0.89154 0.14958

C 1.37523 0.88086 -0.18524

C 0.60756 -0.48584 -0.0443

C 0.57874 2.01667 0.48349

C -0.77103 2.20473 -0.2176

C -1.63847 0.93888 -0.24991

C -0.80337 -0.29589 -0.72975

C -2.38167 0.65199 1.08044

C -3.45507 -0.354 0.64382

C -2.76186 -1.69494 0.32897

C -1.70336 -1.56695 -0.78632

C 3.53903 2.217 -0.33774

C 3.25613 0.73599 1.64522

O 4.9582 -0.41343 -0.37055

H 1.34246 1.09749 -1.26622

O -4.42664 -0.51481 1.67321

C 0.51534 -1.01956 1.40485

H -0.54724 -0.06801 -1.77481

O 3.44022 -2.52958 -0.033

C -3.97908 0.28537 -0.64412

C -2.85516 1.13741 -1.21223

C -5.21197 0.14798 -1.13001

H 1.3144 -1.32249 -1.93981

H 0.99543 -2.56536 -0.71321

H 3.49796 0.02566 -1.74621

H 0.42859 1.8144 1.55124

H 1.13677 2.95642 0.42741

H -1.33758 3.01746 0.25645

H -0.57883 2.52475 -1.25263

H -1.75906 0.26446 1.88844

H -2.86638 1.57006 1.43851

H -2.32577 -2.06667 1.26392

H -3.51759 -2.4285 0.01465

H -2.23558 -1.54678 -1.74429

H -1.09723 -2.47781 -0.81156

H 3.26386 3.05968 0.30417

H 3.23173 2.4545 -1.36358

H 4.63037 2.13296 -0.32043

H 3.03412 -0.25648 2.04343

H 2.70886 1.46908 2.24663

H 4.32528 0.91191 1.79339

H 5.01978 -1.31813 -0.00142

H -4.98642 -1.27231 1.43611

H 1.46037 -1.45992 1.73167

H -0.23126 -1.81542 1.47233

H 0.24991 -0.24897 2.13128

H -3.15175 2.19529 -1.19841

H -2.61035 0.89493 -2.25364

H -5.96239 -0.45258 -0.62009

H -5.52881 0.63995 -2.04703

Conformation 3 of **16**

C 1.39082 -1.55763 -0.87131

C 2.84852 -1.56076 -0.49902

C 3.59085 -0.24981 -0.68394

C 2.91025 0.89063 0.14983

C 1.3761 0.88164 -0.18481

C 0.60717 -0.48444 -0.04464

C 0.58098 2.018 0.48453

C -0.76872 2.20727 -0.21611

C -1.63725 0.94207 -0.24698

C -0.80446 -0.29344 -0.72855

C -2.38157 0.6543 1.08262

C -3.46132 -0.35152 0.63992

C -2.77009 -1.69409 0.32433

C -1.70431 -1.56501 -0.7845

C 3.54101 2.21614 -0.33596

C 3.25697 0.73299 1.64527

O 4.95801 -0.4156 -0.37275

H 1.34341 1.09888 -1.26568

O -4.52522 -0.5323 1.5713

C 0.5151 -1.01951 1.40401

H -0.54925 -0.06584 -1.77392

O 3.43781 -2.53031 -0.03499

C -3.97817 0.28377 -0.64374

C -2.85706 1.14416 -1.20477

C -5.20468 0.12604 -1.1399

H 1.31289 -1.31969 -1.94111

H 0.99324 -2.56368 -0.71586

H 3.49741 0.02611 -1.74708

H 0.43066 1.81538 1.55221

H 1.13997 2.9572 0.42879

H -1.33444 3.02044 0.25819

H -0.57667 2.52712 -1.25132

H -1.75708 0.2663 1.89016

H -2.86609 1.57192 1.44151

H -2.32957 -2.07348 1.25702

H -3.5316 -2.41877 0.0118

H -2.23444 -1.54587 -1.74361

H -1.09749 -2.47552 -0.80763

H 3.26718 3.05817 0.30738

H 3.23328 2.45554 -1.36124

H 4.63226 2.13091 -0.31951

H 2.71027 1.46578 2.2476

H 4.32624 0.90791 1.79359

H 3.03423 -0.25979 2.0423

H 5.01901 -1.32061 -0.00432

H -4.17747 -1.06649 2.3043

H 0.25125 -0.24924 2.13135

H 1.45952 -1.46184 1.72993

H -0.23265 -1.81448 1.47078

H -3.15538 2.20146 -1.18054

H -2.61525 0.91129 -2.24898

H -5.94 -0.49175 -0.63196

H -5.51901 0.61353 -2.06026

Conformation 4 of **16**

C 1.39579 -1.56293 -0.86015

C 2.8538 -1.56029 -0.48912

C 3.59246 -0.24843 -0.68225

C 2.90993 0.89497 0.14578

C 1.37559 0.88046 -0.18766

C 0.60995 -0.48666 -0.03951

C 0.57821 2.01841 0.47621

C -0.77212 2.20138 -0.225

C -1.63741 0.93398 -0.25108

C -0.80176 -0.30242 -0.72519

C -2.38087 0.64722 1.07862

C -3.4555 -0.36645 0.63773

C -2.76111 -1.70513 0.33742

C -1.69944 -1.57593 -0.77399

C 3.53741 2.21916 -0.34798

C 3.25796 0.74643 1.64178

O 4.96048 -0.40885 -0.37174

H 1.34154 1.09174 -1.26966

O -4.4601 -0.63935 1.60841

C 0.51886 -1.01364 1.41225

H -0.54637 -0.08025 -1.77171

O 3.44606 -2.52568 -0.02029

C -3.97501 0.27135 -0.64915

C -2.85535 1.12849 -1.21492

C -5.21333 0.12752 -1.12137

H 1.3166 -1.33164 -1.93132

H 1.0007 -2.56892 -0.69822

H 3.49731 0.02127 -1.74675

H 0.42863 1.82068 1.54488

H 1.13502 2.95859 0.41565

H -1.33974 3.01527 0.2459

H -0.58113 2.51736 -1.26157

H -1.76024 0.26163 1.88907

H -2.86248 1.5698 1.43403

H -2.32615 -2.07107 1.27541

H -3.519 -2.43576 0.03019

H -2.22979 -1.56227 -1.73323

H -1.09012 -2.48479 -0.79414

H 3.2611 3.06442 0.29003

H 3.22925 2.45128 -1.3748

H 4.62887 2.1369 -0.33069

H 2.71244 1.48357 2.23986

H 4.32753 0.92136 1.78795

H 3.03442 -0.24355 2.04518

H 5.02388 -1.31127 0.0024

H -4.96214 0.18304 1.73687

H -0.22961 -1.80733 1.48443

H 0.25631 -0.23903 2.13545

H 1.46343 -1.45466 1.73964

H -3.1544 2.18569 -1.20346

H -2.60881 0.88457 -2.25551

H -5.9389 -0.49387 -0.60186

H -5.54803 0.62339 -2.02974

Conformation 5 of **16**

C 1.3922 -1.55907 -0.86991

C 2.84984 -1.56079 -0.49731

C 3.59093 -0.24917 -0.68289

C 2.90933 0.89146 0.14983

C 1.37524 0.88081 -0.185

C 0.60754 -0.48591 -0.04419

C 0.57882 2.01678 0.48351

C -0.77091 2.20486 -0.21768

C -1.63841 0.93907 -0.24988

C -0.80344 -0.29589 -0.72954

C -2.3818 0.65234 1.08044

C -3.45517 -0.35371 0.6438

C -2.76186 -1.69472 0.3295

C -1.70348 -1.56693 -0.78591

C 3.5391 2.21698 -0.33736

C 3.25611 0.73569 1.64545

O 4.95827 -0.4134 -0.37138

H 1.34255 1.09731 -1.26601

O -4.42716 -0.51414 1.67283

C 0.51539 -1.02006 1.40482

H -0.54734 -0.06827 -1.77467

O 3.43992 -2.52959 -0.03282

C -3.97885 0.28534 -0.64445

C -2.855 1.13758 -1.21232

C -5.21155 0.14731 -1.13067

H 1.3144 -1.32196 -1.93991

H 0.99525 -2.56525 -0.71369

H 3.49761 0.02591 -1.74618

H 0.4285 1.81461 1.55124

H 1.13693 2.95647 0.42742

H -1.33743 3.01768 0.25627

H -0.57868 2.52475 -1.25274

H -1.7593 0.26483 1.8885

H -2.86653 1.57046 1.43838

H -2.32558 -2.06611 1.2645

H -3.5175 -2.42849 0.01544

H -2.23573 -1.54679 -1.74382

H -1.09738 -2.47782 -0.811

H 3.26377 3.05963 0.30451

H 3.23193 2.4545 -1.36325

H 4.63042 2.13294 -0.3199

H 3.03331 -0.25653 2.04377

H 2.70938 1.46928 2.24675

H 4.32537 0.91088 1.79352

H 5.01985 -1.31801 -0.00228

H -4.98368 -1.27483 1.43835

H -0.23139 -1.81576 1.4721

H 0.25025 -0.24962 2.13151

H 1.46037 -1.46073 1.73134

H -3.15172 2.19541 -1.19833

H -2.60997 0.89534 -2.25374

H -5.96175 -0.45341 -0.62062

H -5.5284 0.63886 -2.04789

Conformation 1 of **17**

C -1.47378 1.65236 -0.27094

C -2.81169 1.61587 -0.40602

C -3.61611 0.40755 -0.12732

C -2.92934 -0.93206 0.14318

C -1.41201 -0.83768 -0.22605

C -0.659 0.46587 0.20653

C -0.59094 -2.08996 0.11773

C 0.71181 -2.08583 -0.689

C 1.57834 -0.83847 -0.45308

C 0.72722 0.47556 -0.56058

C 2.39244 -0.89478 0.86561

C 3.44624 0.20623 0.64697

C 2.73674 1.56567 0.74894

C 1.6256 1.72553 -0.30718

C -3.62021 -1.96472 -0.78582

C -3.22735 -1.34854 1.60561

O -4.84407 0.52988 -0.13381

H -1.41636 -0.7786 -1.32528

O 4.50346 0.22663 1.59991

C -0.50331 0.63668 1.74347

H 0.4287 0.53244 -1.61717

O -3.54838 2.70735 -0.76009

C 3.89204 -0.06949 -0.78793

C 2.74413 -0.77203 -1.49459

C 5.10078 0.21245 -1.27438

H -0.97419 2.60377 -0.4359

H -0.37695 -2.14011 1.19246

H -1.15562 -2.99708 -0.12349

H 1.30613 -2.98171 -0.46493

H 0.45571 -2.14342 -1.75742

H 1.82025 -0.73519 1.78067

H 2.88567 -1.87425 0.94527

H 2.34164 1.65758 1.76796

H 3.47698 2.3641 0.61787

H 2.10811 1.97925 -1.2581

H 1.01966 2.60001 -0.0487

H -3.44214 -1.73297 -1.84216

H -4.69937 -1.96025 -0.6126

H -3.24756 -2.97434 -0.59053

H -4.30973 -1.42087 1.75076

H -2.83695 -0.64367 2.34236

H -2.79051 -2.33084 1.81145

H 5.0071 -0.59659 1.48347

H -0.09952 -0.25283 2.23316

H -1.46579 0.86418 2.20654

H 0.15974 1.47636 1.97101

H -4.47955 2.40179 -0.70548

H 3.04371 -1.79469 -1.76253

H 2.44544 -0.2791 -2.42766

H 5.85392 0.69117 -0.653

H 5.38386 -0.02636 -2.29709

Conformation 2 of **17**

C -1.46867 1.65325 -0.27428

C -2.8068 1.61917 -0.40777

C -3.61329 0.41265 -0.12696

C -2.92886 -0.92784 0.14502

C -1.41172 -0.83681 -0.22606

C -0.65579 0.46582 0.20409

C -0.59245 -2.09019 0.11796

C 0.70923 -2.08944 -0.69068

C 1.57912 -0.84396 -0.45663

C 0.72983 0.47162 -0.56405

C 2.39385 -0.90726 0.86168

C 3.44673 0.18639 0.65042

C 2.73786 1.55124 0.74784

C 1.63092 1.71949 -0.31208

C -3.62264 -1.96093 -0.78132

C -3.22638 -1.34138 1.60846

O -4.84099 0.53737 -0.13286

H -1.41734 -0.77889 -1.32533

O 4.47468 0.07586 1.63065

C -0.49861 0.63849 1.74061

H 0.431 0.52808 -1.62053

O -3.54178 2.71158 -0.76244

C 3.89434 -0.07971 -0.78929

C 2.74376 -0.77898 -1.49727

C 5.09174 0.21442 -1.29424

H -0.96736 2.60343 -0.44119

H -0.37728 -2.1397 1.19247

H -1.1591 -2.99653 -0.12157

H 1.30181 -2.98652 -0.46705

H 0.45131 -2.14686 -1.75858

H 1.82037 -0.74984 1.77633

H 2.8899 -1.88388 0.93916

H 2.34075 1.64333 1.76584

H 3.47594 2.35499 0.61665

H 2.11655 1.97078 -1.26188

H 1.02825 2.59647 -0.05454

H -3.44403 -1.73245 -1.83827

H -4.70176 -1.95298 -0.60794

H -3.25285 -2.97108 -0.58338

H -4.30872 -1.41325 1.75408

H -2.83578 -0.63523 2.34387

H -2.78959 -2.32334 1.81602

H 5.02315 0.87558 1.57571

H -0.09268 -0.24967 2.23096

H -1.4609 0.865 2.20443

H 0.16297 1.47976 1.96654

H -4.47348 2.40774 -0.70699

H 3.04291 -1.8025 -1.76263

H 2.44636 -0.28771 -2.43169

H 5.86771 0.67978 -0.68988

H 5.35353 -0.00922 -2.32613

Conformation 3 of **17**

C -1.4679 1.65279 -0.27268

C -2.80596 1.61974 -0.40721

C -3.61355 0.41367 -0.12787

C -2.93028 -0.92745 0.14378

C -1.41271 -0.83736 -0.2258

C -0.65603 0.46455 0.20528

C -0.59485 -2.09144 0.11879

C 0.70747 -2.09147 -0.68859

C 1.57796 -0.84648 -0.45351

C 0.7301 0.46969 -0.56204

C 2.39527 -0.90855 0.86371

C 3.45367 0.18685 0.64476

C 2.74608 1.55344 0.74182

C 1.63087 1.71819 -0.31063

C -3.62391 -1.95936 -0.78398

C -3.22927 -1.34172 1.60668

O -4.84117 0.53922 -0.13469

H -1.41718 -0.77919 -1.32506

O 4.57063 0.12443 1.52829

C -0.49888 0.63614 1.7419

H 0.43154 0.52608 -1.61863

O -3.53984 2.71296 -0.76172

C 3.89358 -0.0785 -0.78914

C 2.74457 -0.78499 -1.49235

C 5.08608 0.23042 -1.2972

H -0.96595 2.60278 -0.43864

H -0.3805 -2.14118 1.19348

H -1.16209 -2.99727 -0.12126

H 1.29948 -2.98874 -0.46422

H 0.45047 -2.14902 -1.75679

H 1.82078 -0.75061 1.7788

H 2.8911 -1.88506 0.94156

H 2.34477 1.65384 1.76003

H 3.48936 2.34874 0.60829

H 2.11312 1.9707 -1.26181

H 1.02773 2.59417 -0.05054

H -4.70318 -1.9509 -0.61159

H -3.25492 -2.96992 -0.58661

H -3.44422 -1.73011 -1.84059

H -2.79355 -2.32423 1.81384

H -4.31179 -1.41268 1.75149

H -2.83849 -0.63638 2.34281

H 4.26773 0.43303 2.39811

H -0.09415 -0.25294 2.2316

H -1.46088 0.8636 2.2059

H 0.16366 1.47664 1.96834

H -4.4718 2.40975 -0.70739

H 3.04488 -1.81009 -1.75052

H 2.44884 -0.29989 -2.43042

H 5.84932 0.70804 -0.68896

H 5.34487 0.00903 -2.33042
